# Screening for Depression, Sleep-Related Disturbances, and Anxiety in Patients with Adenocarcinoma of the Pancreas: A Preliminary Study

**DOI:** 10.1100/2012/650707

**Published:** 2012-05-22

**Authors:** Andrew D. Boyd, Doris Brown, Chris Henrickson, Janet Hampton, Bin Zhu, Farideh Almani, Edgar Ben-Josef, Mark Zalupski, Diane M. Simeone, Jeremy M. G. Taylor, Roseanne Armitage, Michelle Riba

**Affiliations:** ^1^Department of Biomedical and Health Information Sciences, University of Illinois at Chicago, 1919 W Taylor Street MC 530, Chicago, IL 60612, USA; ^2^Department of Radiation Oncology, Wake Forest Baptist Medical Center, Medical Center Boulevard, Winston-Salem, NC 27157, USA; ^3^University of Michigan Hospital Social Work, University of Michigan, 1500 E. Medical Center Drive, Ann Arbor, MI 48109, USA; ^4^University of Michigan Hospital Cancer Center-Pancreatic Clinic, University of Michigan, 1500 E. Medical Center Drive, Ann Arbor, MI 48109, USA; ^5^Center for Human Genetics, Division of Medical Genetics, Department of Medicine, Duke University Medical Center, P.O. Box 3445, 905 LaSalle Street, Durham, NC 27710, USA; ^6^Molecular and Behavioral Neuroscience Institute, University of Michigan, 1500 E. Medical Center Drive, Ann Arbor, MI 48109, USA; ^7^Department of Radiation Oncology, University of Michigan, 1500 E. Medical Center Drive, Ann Arbor, MI 48109, USA; ^8^Division of Hematology and Oncology, Department of Internal Medicine, University of Michigan, 1500 E. Medical Center Drive, Ann Arbor, MI 48109, USA; ^9^Departments of Surgery and Molecular and Integrative Physiology, University of Michigan, 1500 E. Medical Center Drive, Ann Arbor, MI 48109, USA; ^10^Department of Biostatistics, School of Public Health, University of Michigan, 1415 Washington Heights, Ann Arbor, MI 48109, USA; ^11^Department of Psychiatry, University of Michigan, 1500 East Medical Center Drive, Room F6236 MCHC, P.O. Box 0295, Ann Arbor, MI 48109-0295, USA

## Abstract

*Purpose*. Screening for depression, sleep-related disturbances, and anxiety in patients with diagnosed adenocarcinoma of the pancreas. *Materials and Methods*. Patients were evaluated at initial consultation and subsequent visits at the multidisciplinary pancreatic cancer clinic at our University Cancer Center. Cross-sectional and longitudinal psychosocial distress was assessed utilizing Personal Health Questionnaire 9 (PHQ9) to screen for depression and monitor symptoms, the Penn State Worry Questionnaire (PSWQ) for generalized anxiety, and the University of Michigan Sleep Questionnaire to monitor sleep symptoms. *Results*. Twenty-two patients diagnosed with pancreatic cancer participated during the 6-month pilot study with longitudinal followup for thirteen patients. In this study, mild-to-moderate depressive symptoms, anxiety, and potential sleep problems were common. The main finding of the study was 23% of the patients who were part of this pilot project screened positive for moderately severe major depressive symptoms, likely anxiety disorder or a potential sleep disorder during the study. One patient screened positive for moderately severe depressive symptoms in longitudinal followup. *Conclusions*. Depression, anxiety, and sleep problems are evident in patients with pancreatic cancer. Prospective, longitudinal studies, with larger groups of patients, are needed to determine if these comorbid symptoms impact outcome and clinical course.

## 1. Introduction

Pancreatic cancer is an aggressive cancer with a poor prognosis. It is the fourth leading cause of cancer death in the United States [1] with a five-year survival for all stages of 5%. Even for localized, resectable pancreatic cancer, the mean life expectancy ranges from 15 to 19 months [2]. With such a short life expectancy, health-related quality of life is an important aspect of care in these patients. Screening for distress in pancreatic cancer patients is increasingly assessed in clinical trials but has not become a standard component of routine clinical care. The National Comprehensive Cancer Network has implemented clinical practice guidelines in distress management of patients with cancer [3]. In order to incorporate these recommendations into clinical care, knowledge of the prevalence and spectrum of distress and its effect on quality of life in patients with newly diagnosed adenocarcinoma of the pancreas is necessary.

We have recently reviewed the association of pancreatic cancer with depression [4]. Pancreatic cancer is commonly perceived by the public of being a painful and deadly disease, which leads to fear in patients diagnosed with pancreatic cancer that might lead to anxiety and depression [5]. Depression in pancreatic cancer has been reported to have a higher prevalence than in patients with other types of gastrointestinal carcinomas [6]. The range of reported depression varies widely, from 10% to 75%, depending on the instruments used for evaluation and when the evaluation was performed relative to diagnosis and treatment [7–13]. While the link has been known for ages, the relationship is still poorly understood and understudied [14, 15]. Recent studies evaluating patient-reported outcomes list depressive symptoms at 40% for pancreatic cancer [13]. Depression and anxiety may even precede the diagnosis of pancreatic cancer. Anxiety, including presentation with panic attacks, has been reported prior to the diagnosis of pancreatic cancer [16, 17]. An investigation of signs and symptoms of pancreatic cancer by a population-based case-control study found that patients with pancreatic cancer were more likely to report altered ability to sleep than controls (OR, 2.9; 95% CI: 1.3–6.3) [18]. Although pain has been examined as a correlate of depression [19], it appears that there may be a physiologic cause for pain as well [20]. However, the details and spectrum of psychosocial distress and depression in pancreatic cancer patients have not been fully investigated. A recent review confirmed the linkage between pancreatic cancer and depression but was unable to explain the cause or a direct coping style affecting survival [21]. An analysis of 50 inpatient pancreatic cancer inpatients in China revealed that the link between depression and pancreatic cancer is common [22]. A recent population analysis in the Netherlands of 120,852 individuals found a modest decrease in risk of pancreatic cancer associated with past sports activity [23]. However, a prior systematic review did not find a strong evidence of a link with physical activity and pancreatic cancer [24].

A particular association of depression with pancreatic carcinoma has been previously proposed, with Yaskin reporting depression, anxiety, and insomnia as the presenting symptoms for pancreatic cancer as early as 1931 [25]. When compared with patients with gastric adenocarcinoma, pancreatic cancer patients were more likely to fulfill criteria for major depressive disorder within one year prior to diagnosis or at the time of diagnosis [26, 27]. A population-based case-control study found that there was an increase in altered ability to sleep (OR, 2.9, 95% CI, 1.3–6.3) reported in cases compared to controls within 3 years before diagnosis with pancreatic cancer [18]. Additionally, a relationship between depression and pancreatic cancer was found in the general population using a longitudinal population-based study [28] with depression more commonly preceding pancreatic cancer than other malignancies (OR 4.1, 95% CI, 1.05–16.0). Taken together, these data suggest that the relationship between depression, anxiety, insomnia, or sleep related disturbances and pancreatic cancer is likely to be due to more than poor prognosis or fear of pain [5]. The data of this study does reveal a prevalence of the comorbid conditions but does not add additional insight to the cause of that interrelationship.

Another area of recent interest is the relationship between sleep and cancer [29]. The symptom-based approach focuses on the insomnia and daytime sleepiness [29]. A case report of abnormal sleep behavior, similar to sleep-related epilepsy, parasomnia, and night delirium, became a subsequent diagnosis of a tumor in the pancreas [30] which demonstrates how sleep disturbances can precede the diagnosis. Moreover, self-reported sleep disturbances increase during chemotherapy in pancreatic cancer patients [31]. However, the relationship among depression, sleep, and anxiety treatment side effects and response is complex and has not been adequately studied in pancreatic cancer. The objectives of this pilot study were to evaluate the symptoms of depression, sleep, and anxiety disorders in a cross-sectional and longitudinal study in patients presenting to a multidisciplinary pancreatic cancer clinic. In addition, we wanted to evaluate the feasibility of patient's direct entry of data into an electronic data management system. While larger studies have examined a single psychiatric conditions, due to the overlapping of symptoms and possible biological basis of sleep, depression and anxiety this pilot project were initiated to evaluate the co-morbidities with pancreatic cancer. 

## 2. Material and Methods

Over a six-month study period, 27 patients presenting to the University of Michigan Multidisciplinary Pancreatic Cancer Clinic were approached to participate in the study, which was approved by the University of Michigan Institutional Review Board. The exclusion criteria were inability to communicate, understand verbal or written English. Individuals were approached during their initial consultation appointment by the project coordinator. As this study was designed to be conducted in conjunction with the clinic visit, a number of stipulations were imposed on this study by both the Comprehensive Cancer Center and the Internal Review Board to protect the patients. The treating clinical providers were the ones who referred the patients to the research staff. Twenty-four patients provided written informed consent and completed at least one assessment. A total of 3 people were approached and turned down the research team, and two other patients without pancreatic cancer were not included in the study. 22 research subjects subsequently had confirmed pancreatic cancer, who were included in the analysis. The Personal Health Questionnaire 9 (PHQ9) [32] was utilized to screen for depression and to monitor symptoms during the course of care. Subjects completed the Penn State Worry Questionnaire (PSWQ) [33] for generalized anxiety. The University of Michigan Sleep Assessment Questionnaire was utilized to monitor sleep symptoms. Patients were evaluated at their intake consultation and at subsequent follow-up visits during the study period not frequently than once a month for the complete duration of the 6-month project. Any patient who screened positive for depression, sleep disorder, or anxiety had clinical followup by the clinic physician, a visit by the clinical social worker, or a referral to a psychiatrist. In addition, all patients received information about the psychooncology program at the cancer center. One goal of the study was to repeat the measurements over time. Since the same psychometric surveys would be conducted multiple times, a restriction of completing all of the tests within 30 minutes was imposed. Other psychometric tests were not permitted due to the length of time to complete the alternative psychometric tests. During the study, the research team was restricted to approaching the research subjects to once a month so as not to burden the research subjects.

In order to facilitate collection of data and incorporation into an electronic record, subjects initially utilized a tablet personal computer with export of the data into the M-STRIDES clinical server [34]. This server was designed for intake screening for the Department of Psychiatry and also contains a number of psychometric surveys which are patient administered for longitudinal followup. The research subjects were told that the survey data would become part of the clinical record. If there was difficulty with data entry by subjects, hard copy questionnaires were utilized by research subjects, and the data was subsequently entered by research assistants. 

### 2.1. Statistical Analysis

Descriptive statistics are used to summarize the baseline data. Spearman's correlation coefficient is used to assess association between pairs of factors. Associations between the three psychometric scores and stage of pancreatic cancer were assessed using Kruskal-Wallis tests from analysis of variance (ANOVA). A visual examination of repeat psychometric tests was performed.

## 3. Results

Demographic characteristics of the subjects enrolled in the study are presented in Table 1. The mean age was 66 years, and there was a nearly equal male and female ratio.

See Table 2 for a listing of the stages of pancreatic cancer. Of the complete sample, two did not have pancreatic cancer, one did not have a cancer diagnosis, and the other had B-cell lymphoma.

### 3.1. Screening for Depressive Symptoms

Subjects completed the PHQ-9 questionnaire to evaluate for depressive symptoms and monitor the symptoms over the course of treatment. The PHQ-9 is a series of nine questions with four categories of frequency at which subjects experience symptoms. The responses to the nine questions were summed for a score ranging from 0 to 36. Results from the PHQ questionnaire are shown in Table 3. A score of 0–5 indicates no major depressive symptoms, a score of 6–9 mild major depressive symptoms, scores between 10–14 for moderate depressive symptoms, while a score of 15–19 moderately severe major depressive symptoms. However, 68% of the research subjects had mild or worse major depressive symptoms. There were no individuals with a score of greater than 20, which would indicate severe major depressive symptoms. With 33% of patients having moderate or moderately severe depressive symptoms this would suggest that the largest groups of research subjects have mild depression.

### 3.2. Screening for General Anxiety

The Penn State Worry Questionnaire (PSWQ) was utilized to assess anxiety in our population of pancreatic cancer patients. The Penn State Worry Questionnaire is a series of fifteen questions in which respondents indicate the frequency of worrying over the past week on a scale of 0–6. The responses are summed to a score ranging from 0 to 90. Results from the PSWQ are shown in Table 3. A score of 40 or less is seen in individuals with subclinical anxiety, scores from 40 to 60 indicate moderate anxiety of possible clinical significance, and a score of greater than 60 indicates a likely presence of anxiety disorder. 2 individuals had a likely anxiety disorder with large percentage of patients demonstrating some symptoms of anxiety.

### 3.3. Screening for Sleep Disturbance

Sleep problems were assessed utilizing a University of Michigan Sleep Assessment Questionnaire (SAQ). This questionnaire assesses the frequency of disturbance in participant sleeping patterns over the past month. The scale provides a total summed score from 0 to 26. 3 research subjects demonstrate the symptoms of a possible sleep problem. See Table 3 for additional information.

### 3.4. Interrelationship between Measures

Using Spearman correlation, the PHQ is trended with the SAQ (Spearman's rho = 0.56, *P*-value = 0.09), which is not quite statistically significant at the 0.05 level that is suggestive of more depressive symptoms in those with more severe sleep problems. We estimate that 16% of the depression score (PHQ) variance is explained by SAQ using ANOVA. PHQ scores were not correlated with PSWQ worry scale (Spearman's rho = 0.02, *P*-value = 0.97). SAQ did not correlate with PSWQ (Spearman's rho = 0.12, *P*-value = 0.60).

Sleep problems from the SAQ trended with stage of cancer but were not statistically significant (Kruskal-Wallis statistic = 5.15, *P*-value = 0.08). The PHQ also trended (Kruskal-Wallis statistic = 4.34, *P*-value = 0.11), and PSWQ was not associated with stage (Kruskal-Wallis statistic = 1.46, *P*-value = 0.48). There was no association of any of the three scores with age.

The tables are a description of the data on initial enrollment into the study. Research subjects once enrolled in the study, all three psychometric tests were to be performed no more frequently than once a month during follow-up appointments. Since the follow-up evaluations were only performed on 13 research subjects, formal analysis of the data was not performed. Visual examination of the trends over time in the three tests did not reveal any suggestive patterns. However, one research subject who was moderate MDS became moderately severe MDS during the study. All three measures were plotted over a consistent time interval separately with the initial survey being zero. The study was also a complete failure in encouraging the use of tablets by patients. By examining the audit logs, only 10% of the patients entered their own data into the M-STRIDES system. The other 90% filled out the paper forms, and the data was entered by the research staff.

## 4. Discussion

The main finding of this preliminary study is 23% of the patients who were part of the pilot project and had symptoms of either moderately severe major depressive symptoms, likely anxiety disorder, or a potential sleep disorder at some point during the study. This number is calculated from the four individuals who appear in Table 3 (one moderately depressed, one likely anxiety disorder, and two sleep problem) and one individual becomes moderately depressed during the longitudinal evaluation. Another two individuals screened positive sleep disorders and anxiety at follow-up appointment. With prevalence of symptoms, this high, routine screening and concern for patients with these illness are appropriate. Pancreatic cancer has a very poor prognosis and a reputation as being one of the deadliest [35]. Depression, anxiety, and insomnia have been shown to be comorbidities in cancer patient populations, and screening has been recommended to improve the overall care of patients [36]. A number of patients also had lower severity of symptoms. While a psychometric survey does not replace a trained clinicians, evaluation, when patients present with this level of symptoms, additional investigation is warranted to clarify the cause.

Another finding from this study which is not represented in the statistical outcome is the routine collection of psychometric data in a busy multidisciplinary pancreatic cancer clinic. A large percentage (90%) of patients were not willing to use the tablet PC; the routine collection of psychometric data was accomplished through the traditional methods of pen and paper. Concerns about the validity of the measurement being conducted on the tablets are not founded in this study; however future research is warranted. Future trials should consider the challenges of using tablet PCs and have alternative methods for a successful project. Most cancer centers have patient education material on psychooncology, and referrals are common; however the use of psychometric surveys is a more proactive approach for screening for acute mental health disorders. Obviously the complex nature of the interaction between the cancer, knowledge of the diagnosis, side effects of chemo and radiation therapy, and previous history of mental will not be revealed in a simple psychometric survey; the data can help inform and engage patients and clinicians to address concerns and seek out additional services. While current oncology guidelines focus on distress management [3], the nature of anxiety, depression, and sleep disorders may not be revealed by focusing on distress. Future studies should evaluate the differences between the distress thermometer [3] and other Psychometric tests.

Sleep disturbance accounted for 16% of the variance in depressive symptoms. Sleep was also more disturbed in those with a more advanced stage of cancer. If, as we believe, that poor sleep contributes to both poor quality of life and increased depression in cancer patients, then there is good reason to pursue improving sleep in these patients. Future studies should be significantly powered to better describe the relationship among these variables and to explore strategies for improving sleep. Behavioral interventions have been shown to be effective in improving sleep in other cancers [37] and are particularly appealing in patients already undergoing rigorous treatment.

Compared to previous studies, the individual prevalence of anxiety, depression, and sleep disorders appears to be lower than previously reported [4, 6–11, 13, 26, 38–42]. While the number of participants is large in comparison to historical reports of pancreatic cancer and depression, the numbers are insufficient for a robust statistical analysis. Other limitations were encountered in this study. First, the pancreatic cancer clinic at University of Michigan is a referral location, so the initial diagnosis is often made prior to the patients visit to the clinic. Second, a study that would verify the claim of the presence of depression before pancreatic tumor diagnosis would necessitate psychometric screening be performed prior to the malignant diagnosis. Third, part of the research protocol may have created opportunity for participant exclusion. The treating clinicians evaluated the potential research subjects for suitability for inclusion into this study. If a patient was severely depressed or anxious, an immediate referral to psychooncology would have been made, and the research staff may not have had access to the patient for study participation. A number of different reasons, that this requirement was included, pertained to cases where the patient just recently told of their diagnosis with cancer or the patient was despondent. As the treating clinician had the therapeutic relationship with the patient, the study determined they were in the best position to evaluate; this was a requirement for working in the clinic. Fourth, no information was collected about the current use of psychotropic drugs by the patients or the recommendations for cancer treatment received by the subjects. Additional data points about historical depression, anxiety, and sleep disorders or other health-related events were not recorded.

In a larger retrospective series of 258 patients with pancreatic cancer, depression did not affect survival, although it was common with 54 out of 258 patients (21%) with a diagnosis and treatment for depression at the time of the pancreatic cancer diagnosis [43]. The median survival time for depressed patients was 4 months compared with non-depressed patient's median survival time of 5 months (not significant). There was also no association of cancer stage to the presence of depression. These findings are in agreement with our findings of a lack of major depressive symptoms, anxiety, and sleep disorders in patients presenting to a dedicated pancreatic cancer clinic. However, retrospective diagnosis of depression can lead to lower prevalence diagnosis. Our prospective trial does shed some light on how sleep, anxiety, and depression symptoms are present using objective psychometric instruments. The lack of statistical correlation with survival does not reduce the need to evaluate and treat the symptoms in patients.

It is possible that the decrease in reported psychosocial distress in ours and in Kelsen's studies reflects changes in attitudes among pancreatic cancer patients. Another explanation is that prior reports did not utilize a prospective, standardized screening which was utilized here. An alternate explanation may be that in both of our studies patients presented to a comprehensive cancer center and were therefore motivated to seek care, which may suggest bias in our patient population. Additional study is warranted to further evaluate the prevalence and the correlation to improve the lives of patients diagnosed with pancreatic cancer. Future plans to design a larger prospective study examining the correlation between the three comorbidities and pancreatic cancer with comparison against a control group are needed. Additional data collection about the treatment modalities and prior mental health history will add additional insight into this complex relationship.

## Figures and Tables

**Table 1:** Demographic characteristics of study subjects.

Characteristic	
Gender	
No. of males	12
No. of females	10
Ethnicity	
No. of Caucasians	20
No. of African-Americans	1
No. of unknown	1
Age (years)	Mean 65; range 52–83

**Table 2:** Stage of pancreatic cancer.

Stage of pancreatic cancer	Number of study subjects	Percent
Resectable	6	27.27
Locally, advanced, unresectable	7	31.82
Metastatic	9	40.91

**Table 3:** Prevalence of depressive symptoms, general anxiety, and sleep disorders in pancreatic cancer patients. MDS: major depressive symptoms.

Depressive symptoms category	Number of patients	Percentage of patients
0–5: no MDS	9	41
6–9: mild MDS	7	32
10–14: moderate MDS	5	23
15–19: moderately severe MDS	1	5
20–36: severe MDS	0	0
General anxiety symptoms category		
Data not available	1	5
<40: subclinical anxiety	12	55
40–60: moderate anxiety of possible clinical significance	8	36
>60: likely anxiety disorder	1	5
Prevalence of sleep disturbance		
Data not available	1	5
0–10: no sleep disturbance	10	45
11–26: potential sleep problem	9	41
>26: sleep problem	2	10
